# Pressure Anaetshesia

**Published:** 1904-08

**Authors:** G. B. Speer

**Affiliations:** Youngstown, O.


### PRESSURE ANAETSHESIA.

By Dr. G. B. Speer, Youngstown, O.

(Written for *The American Journal of Dental Science.*)

This subject is of very great importance to the profession at the present time as I believe that the removal of the nerve and the filling of roots properly is one of the most needed of the operations we use at this time. I first attempted the removal of the nerve by using cocaine and vulcanite by pressure and while success can be attained by that method it is far from satisfactory in my hands, so I took up the use of the syringe and there I found the trouble lay in the getting of a point. First I used just a taper point and a taper burr that was good in some cases, but was of no use in case of exposure, so I made a point with a bell shaped end in which I inserted a short piece of rubber hose about one-eighth of an inch in diameter that worked very well in case of large exposure, but was useless in small teeth. Then I reversed the procedure and made a point with a shoulder for the rubber to hold against and I can get that into small exposures. I then found that I needed those different points bent to several different angles and so made a point out of a long needle which you can get at a drugstore—about two inches long—and use this in a root where the nerve is dead except a small piece at the point of some roots. I now have a very good supply of points but am liable to make some of other shapes at any time. I then turned my attention to getting better control of my syringe, and made an attachment by which I use screw power and I am very well satisfied with the way in which I can control the pressure, and that is a point not to be overlooked. Next, do not forget to use the rubber dam for I use a saturated solution of cocaine in distilled water and only a small amount is necessary. Aonther essential point is to insert your broach to the end of the root by a straight push, then rotate till you have twisted the nerve off before you attempt to withdraw it and you will not have so much hemorrhage to contend with. I do not think any one will ever go back to the use of arsenic if they try this method. It leaves the tooth in so much better condition, as the canals are left smooth and clean and less time is required in which to do the work. I have frequently finished a tooth at the first sitting when the patient came from a distance, making it an object for them as well as for myself. You also get rid of the patient who comes in to have a tooth treated and never comes a second time. This treatment gives a tooth less chance to become discolored as it can be kept dry during the operation. I have been using this mode of procedure for the past two years and believe I am justified in claiming to be successful in my work, and would be glad to answer any questions by personal letter, or preferably, through the journal, as others may read the same.

---

### LIQUID TO USE IN PORCELAIN WORK.

By J. L. Helmer, D. D. S.,
Kokomo, Ind.

(Written for *The American Journal of Dental Science.*)

